# Research progress and trends of insect high-temperature stress: insights from bibliometric analysis

**DOI:** 10.3389/finsc.2025.1625155

**Published:** 2025-07-30

**Authors:** Jiapeng Li, Xianting Wang, Rui Guo, Zikun Li, He Lv, Yiping Wang

**Affiliations:** ^1^ Country College of Forestry and Biotechnology, Zhejiang Agricultural and Forestry University, Lin’an, Zhejiang, China; ^2^ Conservation and Utilization Section, Anji Hynobius National Nature Reserve Management Center, Huzhou, China; ^3^ Science Monitoring Office‌, Administration of Zhejiang Qingliangfeng National Nature Reserve, Hangzhou, China; ^4^ College of Life Sciences, Huzhou University, Huzhou, China

**Keywords:** bibliometrics, global warming, insect, climate change, hot spots and frontiers

## Abstract

Climate warming, particularly extreme temperature events, poses a major threat to insect survival and diversity. Thus, understanding insect responses to high temperatures is increasingly important for predicting their resilience and distribution under climate change. In this study, we analyzed 4,417 articles from the Web of Science Core Collection using VOSviewer, CiteSpace, and the Bibliometrix R package. The results show that since 2012, the number of publications on insect responses to high temperatures has been increasing year by year at an average annual growth rate of 3.5%, reflecting the increasing research interest in this field. Secondly, the response of insects to high temperatures is a multidisciplinary research field. The PLoS One journal has the largest quantity of published articles, boasting the highest total citations and H-index globally. Hoffmann AA and Du YZ are the most productive authors. Furthermore, keyword analysis revealed research focus on molecular responses such as gene expression and heat-shock proteins. Cluster analysis of bibliographic coupling identified 15 major research themes, results demonstrate that the field evolved from describing physiological traits to dissecting biochemical and reproductive mechanisms underlying thermal tolerance, aiming to uncover biological responses and adaptive strategies under heat stress. To summarize, this study provides an overview of current research trends and emerging priorities in insect heat stress biology.

## 1 Introduction

As ectothermic arthropods, insects are highly sensitive to ambient temperature changes that affect survival, distribution, and population dynamics (1–3). The global average surface temperature has increased by 1.1°C since the Industrial Revolution, with a projected rise of 2–5°C anticipated by the end of the 21st century (4). Besides raising average global temperatures, climate change increases the frequency and severity of extreme events such as heatwaves (5, 6). These events influence insect physiology, behavior, life cycles, distribution, and ecological interactions (2, 3, 7). Given these impacts, recent studies have examined how insects cope with rising temperatures and heat extremes at individual and ecosystem levels.

Insect responses depend on heat tolerance and adaptation capacity (3, 8, 9). Thermoreceptors in the peripheral nervous system detect temperature changes and signal the central nervous system, triggering behavioral and physiological responses such as metabolic regulation, heat absorption, hormonal changes, and heat shock protein (HSP) expression (1, 10–17). For example, *Drosophila melanogaster* thermosensitive neurons detect temperature changes as small as 0.5°C, while *Atta vollenweideri* ants can sense shifts as slight as 0.005°C (18, 19). In response to increased environmental temperature, insects use behavioral (e.g., ventilatory control, evaporative cooling) (1, 17), biochemical (e.g., increased HSP expression) (20, 21), and neurochemical strategies (e.g., biogenic amines like octopamine enhancing heat tolerance) to manage heat stress (15, 22, 23). For instance, *Nilaparvata lugens* shows improved heat tolerance linked to HSP overexpression in long-winged morphs (20, 21). While numerous studies have explored how insects respond to high temperatures, tracking trends in insect heat stress research is challenging due to variation in focus, timing, and data sources. Bibliometric analysis quantifies publication trends, identifies key topics, and maps research networks. It is therefore imperative to undertake a comprehensive quantitative analysis of the existing literature to provide a detailed and objective summary of the research development and trends in insect responses to high temperatures.

Bibliometric analysis is a mathematical and statistical research method for quantifying scientific production and impact, which is used to analyze and visualize critical features of published articles and identify research trends in a given field through literature databases (24–26). This study aims to offer a structured and impartial perspective on the advancement of scientific inquiry regarding how insects react to elevated temperatures. This study applies bibliometric tools to analyze 4,417 articles on insect responses to high temperatures, assessing publication trends, author collaborations, keyword patterns, and thematic clusters to understand research evolution and future directions. The objective is to examine both domestic and international research trends on insect responses to high temperature, the current research focal points and the prospective trajectory of this research domain, with a view to offering insights and guidance for the future advancement of research on insect adaptation to high temperature and the protection of beneficial insects.

## 2 Materials and methods

### 2.1 Data collection and screening

A thorough database of research investigating the effects of high-temperature stress on insects is essential for conducting a bibliometric analysis. Currently, the only database that contains comprehensive details on cited references is the Web of Science Core Collection (WoSCC https://webofscience.clarivate.cn/wos/), which is extensively utilized in numerous bibliometric analyses (27–29). Therefore, we used the WoSCC to construct a bibliographic database on the topic of insect responses to high temperatures. Specifically, search strategy: (1) Topic = [(“heat stress” OR “high temperatures” OR “thermal stress” OR “heat conditions” OR “heat waves” OR “heat shock”) AND (“insect” OR “Diptera” OR “Coleoptera” OR “Hemiptera” OR “Lepidoptera” OR “Hymenoptera” OR “Blattodea” OR “Isoptera”)] (These insect orders based on their diversity, ecological, and economic importance). (2) Language = “English”. (3) Article type = (“article” OR “review”). (4) Time = January 1, 1996 to September 2, 2024. After manually filtering the search results to remove irrelevant articles, we obtained a total of 4,417 articles that met the search criteria for bibliometric analysis. Finally, all articles were exported in the form of full records, including cited references, and the exported records were saved as plain text files.

### 2.2 Bibliometric analysis

The bibliometric analysis tool utilized in this research aligns with the one applied in earlier studies (29, 30). VOSviewer (version 1.6.20) was used for network visualization (31), CiteSpace (version 6.3.R1 64-bit) for detecting citation bursts and temporal patterns (32, 33), and Bibliometrix R package (Version 4.0.0) for statistical analysis (34, 35).

The Bibliometrix R-package offered a range of innovative and distinctive tools for quantitative investigation within the fields of bibliometrics and scientometrics (34). These tools could be utilized to perform various bibliometric analyses, such as those pertaining to publication metrics, scientific output, citation references, generation of word clouds, and analysis of trending topics (29). VOSviewer was a software program developed by researchers at Leiden University in the Netherlands for the analysis of literature and the visual representation of knowledge (31). The software was open source and enabled the user to examine the intricacies of research problems efficiently through the utilization of a range of features, including clustering, labelling, and density views (30). In this study, CiteSpace and Bibliometrics were used to calculate the total number of publications and annual publications, mean total citations per paper, mean total citations per year, single country publications (SCP), multiple country publications (MCP), H-index, total local cited references, and total citation score of local global. We computed centrality and sigma values. Centrality values indicate a node’s importance and influence in a network, while sigma values reflect the strength of connections between nodes. These values were employed to evaluate influential journals, authors, countries, and academic institutions, as previously described (28, 30). The CiteSpace and VOSviewer software and the Bibliometrix package were employed for the analysis and visualization of the co-citation network of author, country and institution, in addition to the co-occurrence network of keywords. The bar charts of publication counts were generated by Graphpad Prism (Graphpad Prism 9.3.0, Graphpad Software, USA). Furthermore, bibliographic coupling networks were constructed using the Bibliometrix package. Bibliographic Coupling Network: A network where nodes represent documents, and links indicate shared references, showing how documents are related through common citations.

## 3 Results

### 3.1 Dataset overview

**Figure 1:**
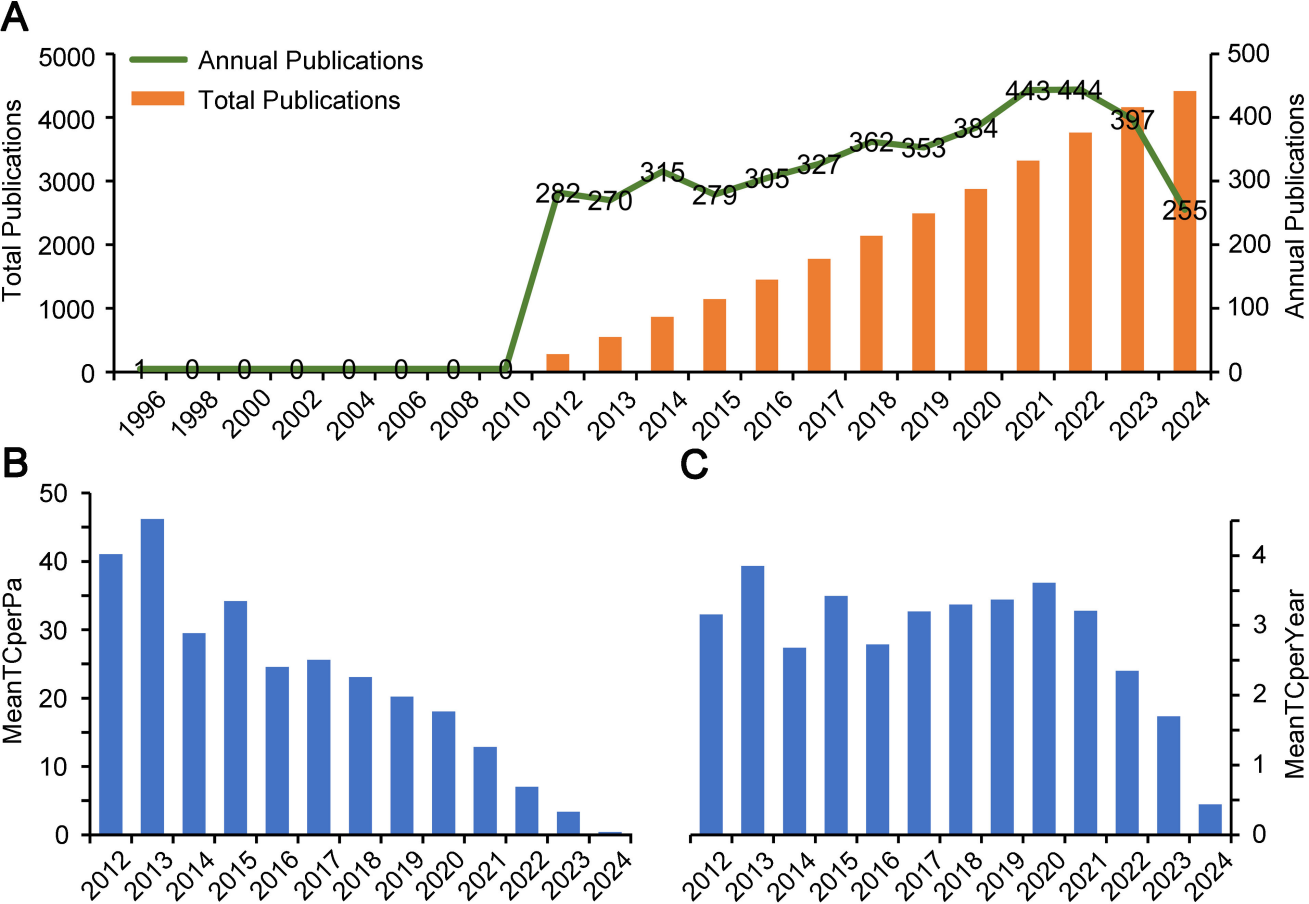
Publication status in the field of insect response to high temperatures stress. **(A)** Timeline of total publications and annual publications in the field of insect response to high temperatures stress from 1996 to 2024. **(B)** The average total number of citations per paper from 2012 to 2024 (MeanTCperPa, which stands for Mean Total Citations per Paper). **(C)** The average total number of citations per year from 2012 to 2024 (MeanTCperYear, which stands for Mean Total Citations per Year).

Over the 30 years from 1996 to 2024, a total of 4417 articles have been published in the field of insect responses to high−temperatures. **Figure 1A** shows a marked increase in publications after 2012, while the number of publications fluctuated, it overall maintained a steady growth trend, with an average annual increase of 3.59%, indicating growing research attention. For the citations of papers, both the total number of citations per article and the average citations per year have decreased, this could be due to shorter accumulation time rather than reduced interest (**Figures 1B, C**). The peak mean value of global total citations occurred in 2013, reaching 46.21 times, while the lowest was recorded in 2023 at only 3.40 times (**Figure 1B**). The highest mean total citations were also noted in 2013 (3.85 times) and 2020 (3.61 times), whereas the lowest average total citation was just 1.70 times in 2023 (**Figure 1C**). Data for 2024 is not included as the year is not yet complete.

**Figure 2:**
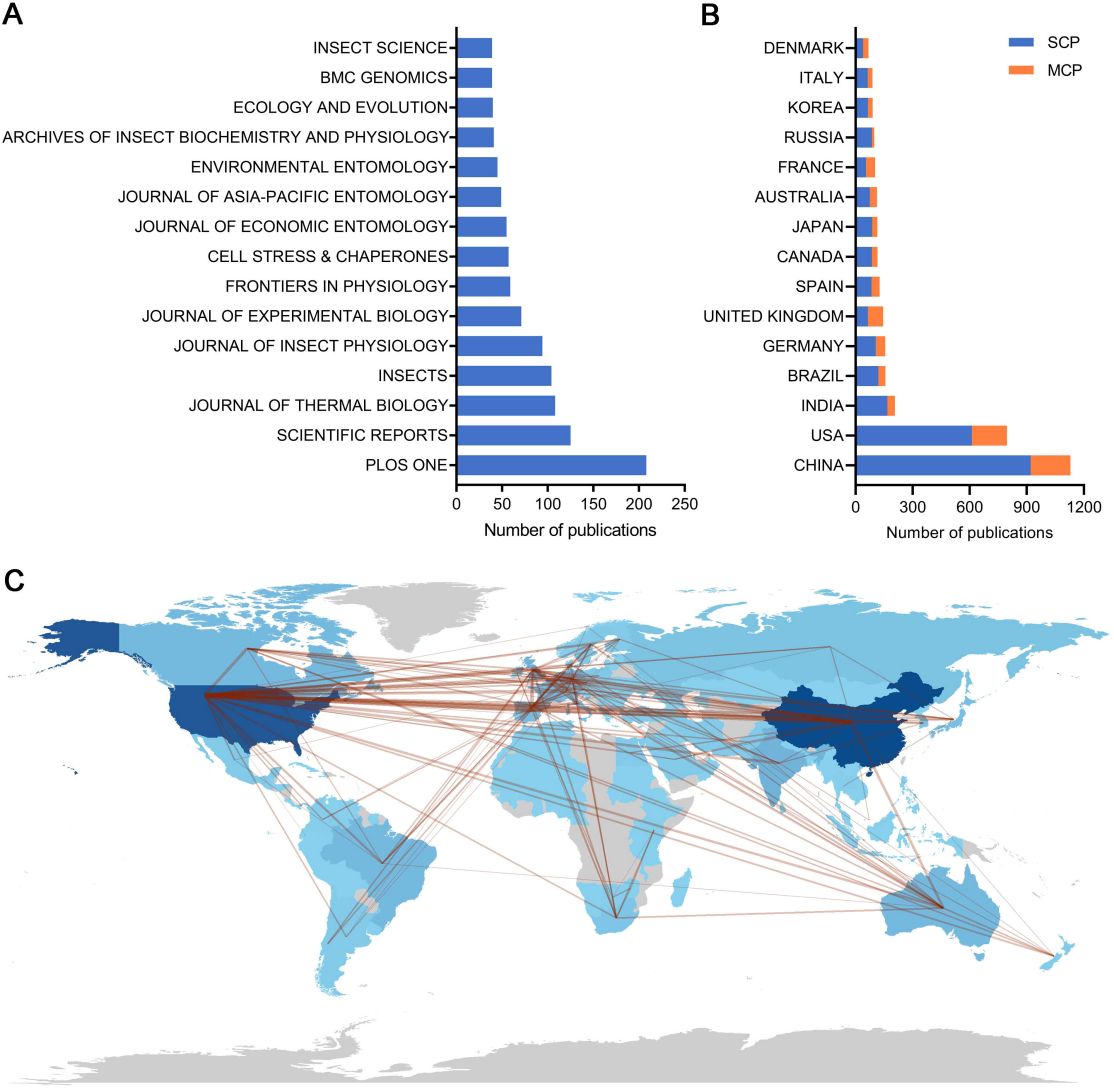
Leading journals and country scientific production in insect response to high temperatures during 2012–2024. **(A)** number of publications for the top fifteen leading journals. **(B)** The number of publications from the top fifteen countries and the distribution status of SCP (Single country publications) and MCP (Multiple country publications). **(C)** The Country collaboration network is represented, where the darkness of the color for each country is proportional to the number of publications, with darker shades indicating a higher number of publications. Straight lines indicate the number of collaborative publications between different countries.

This multidisciplinary literature dataset spans across multiple disciplinary fields, including Zoology (3,051), Environmental Sciences Ecology (2,856), and Biochemistry Molecular Biology (2,775). A total of 847 different sources were identified in the literature, with the ten most frequently publications presented in **Figure 2A**. The top-ranking journal was PLoS One, which published 208 articles with 6,397 citations. While Scientific Reports, Journal of Thermal Biology and Insects with 125, 108 and 104 articles with 2,508, 2,223, and 801 citations published followed closely behind, respectively (**Figure 2A**, **Supplementary Table S1**). The PLoS One journal also ranks first, with an H-index and a G-index of 41 and 61, respectively (**Supplementary Table S1**). However, Proceedings of the National Academy of Sciences (PNAS) is the most cited journal (7,187 citations) with 33 articles and a g-index of 26. These results demonstrate that the journal of PLoS One is an important journal in the field and that PNAS is becoming increasingly important.

To gain a deeper understanding of the phenomenon of authorship and collaboration in the field, an analysis was conducted on three key factors: country, institution, and author. As shown in **Figure 2B**, our analysis shows that China and the USA are the two countries with the largest number of scientific publications, with 1,129 and 796 articles, respectively, corresponding to a cumulative share of 43.6%. Nevertheless, neither country is in the highest position concerning the average article citation index, with China having a relatively low score of 14.6 (**Supplementary Table S2**). For the proportion of Multiple country publications (MCP), our analysis shows that all publications have an overall MCP below 20%. Specifically, the two countries with the highest output of scientific research, namely China and the USA, exhibited MCP ratios below the average, at 18.5% and 23.2%, respectively (**Figure 2B**, **Supplementary Table S2**). In contrast, the countries with a relatively limited number of articles, namely the United Kingdom, Belgium, South Africa, France and Denmark, exhibit higher MCP rates, with 55.2%, 51.6%, 50.0%, 47.1%, and 42.6%, respectively (**Figure 2B**, **Supplementary Table S2**). The percentage of MCP that a country contributed is indicative of the degree of international collaboration in the country, our research findings suggest that there is potential for enhancement in international collaboration within the field. The framework of national collaborations is illustrated in **Figure 2C**, which depicts China, the USA, India, Brazil, and Germany as the primary actors within this network. Analyses of research institutions reveal similar trends to countries, with five of the top 10 institutions coming from China, two from the USA, and one each from France, Denmark, and Russia (**Supplementary Table S3**).

**Figure 3:**
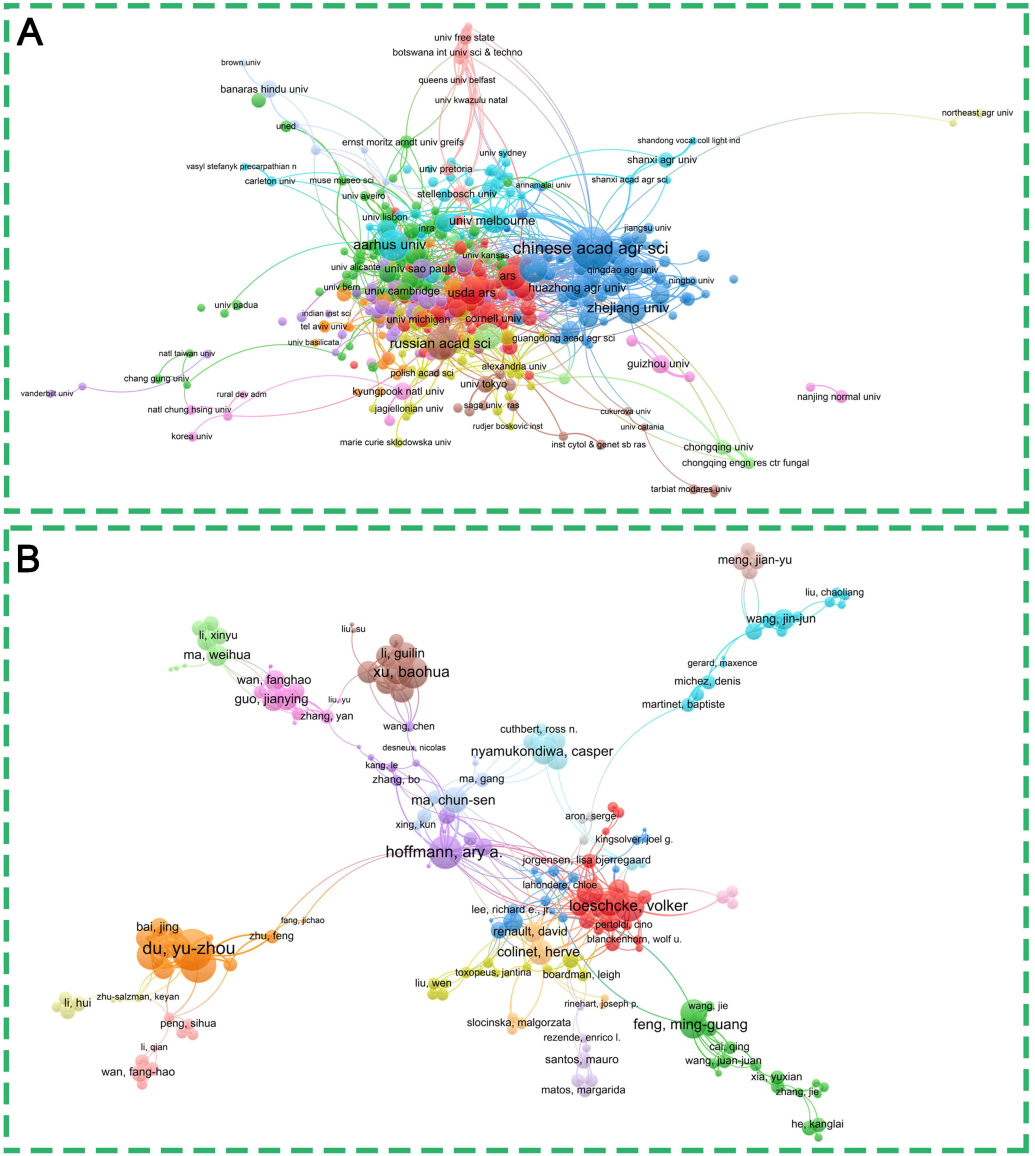
Collaboration networks and their corresponding clusters: **(A)** showing connections between institutions, and **(B)** illustrating connections between authors, both identified using VOSviewer. Each node represents an institution or author that meets the filtering thresholds; node size is correlated with their publication numbers, and the curved lines represent the number of collaborative publications between different institutions or authors. The color of an element indicates the cluster it belongs to, and different clusters are indicated by different colors.

Chinese Academy of Agricultural Sciences ranked first with 200 articles contributed with had a low centrality score of 0.05 (**Supplementary Table S3**). Except for the University of California system, which published 195 articles with a centrality value of 0.33, the centrality of the rest of the institutions was low (**Supplementary Table S3**). The higher the centrality, the more central and influential the institution is in the cooperation network; the lower it is, the more marginal and less influential the institution is. This suggests that the current connections among institutions are not tight enough, the collaboration between the various institutions needs to be deepened. Institutions should engage in more collaboration, including inter-institutional and international partnerships. The cooperation network of relevant institutions is shown in **Figure 3A**, with the Chinese Academy of Agricultural Sciences, the University of California, and the National Center for Scientific Research (CNRS) as significant positions.

We included a total of 299 authors and established a collaborative network, as shown in **Figure 3B** that authors from the same research institution show stronger collaborative relationships. The ten authors with the highest productivity and greatest influence in the field of insect responses to high temperatures are presented in **Supplementary Table S3**. Du YZ and Hoffmann AA. are considered the most influential authors in the field of insect responses to high temperatures, having published 46 and 43 research papers on the topic, respectively. Additionally, Hoffmann AA. is the top-ranked author globally in terms of h-index, g-index, and total citations (**Figure 3B**, **Supplementary Table S3**). Also, to provide a more intuitive representation of the affiliations and countries to which authors belong, Sankey diagrams are employed to visualize the relationships between authors, affiliations and countries (**Supplementary Figure S1**).

**Figure 4:**
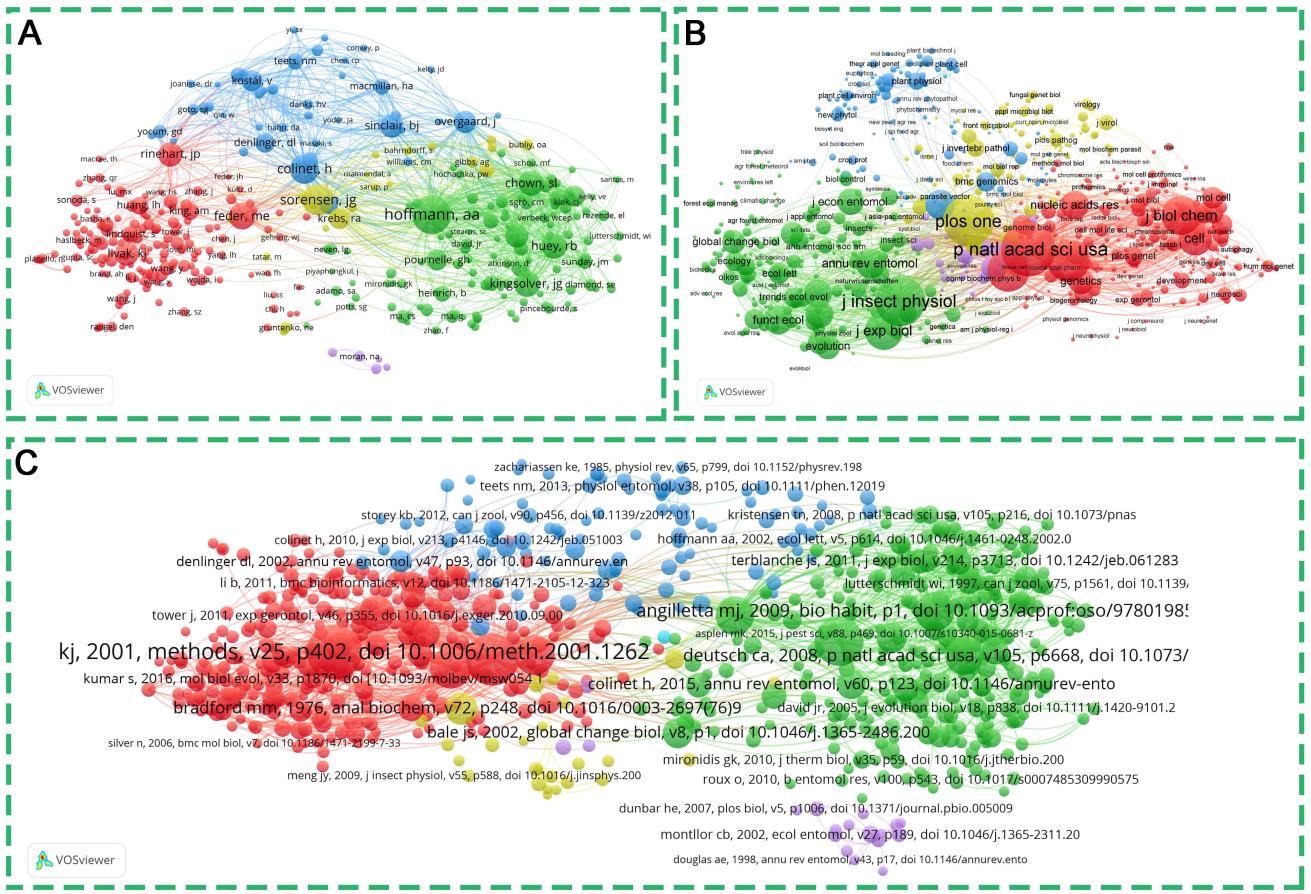
Network visualization of co-citation. **(A)** Network of author co-citations; **(B)** Network of journal co-citations; **(C)** Network of co-cited references. The color of an element indicates the cluster it belongs to, and different clusters are indicated by different colors.

To achieve a more comprehensive understanding of the influential authors, journals, and references within the field, a co-citation analysis was performed, as illustrated in **Figure 4**. The visual network of co-cited authors is presented in **Figure 4A**. The author with the highest number of citations is Hoffmann, AA, with 1,009 citations, followed by Sorensen, JG, Colinet, H, Chown, SL, and Sinclair, BJ, with 687, 626, 487, and 427 citations, respectively (**Figure 4A**, **Supplementary Table S4**), indicating their relative popularity. Additionally, we found that there is no significant correlation between centrality and co-citation counts, indicating that the indication of international influence may not be directly proportional to the strength of a partnership. **Figure 4B** and **Supplementary Table S5** showed cited journals and frequency counts, PNAS (7,187 citations) was the top-ranked journal, followed by PLoS One (6,403 citations), Journal of Insect Physiology (6,177 citations), Nature (4,595 citations), and Science (4,621 citations). In addition, 700 references (the minimum number of which was 20) were collated from 4,417 publications to analyze the co-cited references (**Figure 4C**). Among them, the most-cited reference was published by Feder M.E., et al. (395 citations); this publication demonstrated the essential function of Heat Shock Proteins in the response of organisms to stress (36) (**Figure 4C**, **Supplementary Table S6**). The second reference was published by Hoffmann, A.A., and has been cited 223 times (**Figure 4C**, **Supplementary Table S6**), this study reports how Drosophila research has been used to understand the evolution of plastic responses, trade-offs, and limits to selection. It also establishes connections between laboratory research and the evolutionary mechanisms that give rise to population and species differences (37). In brief, the most frequently cited works tend to be the most actively discussed in the context of citation bursts.

### 3.2 Identification of knowledge base and research frontiers

According to the theoretical framework of research fronts, the research fronts in a certain field represent all documents published within a certain period of time (33). The emergence of salient topics is discerned through an examination of keywords across all published documents (29).

**Figure 5:**
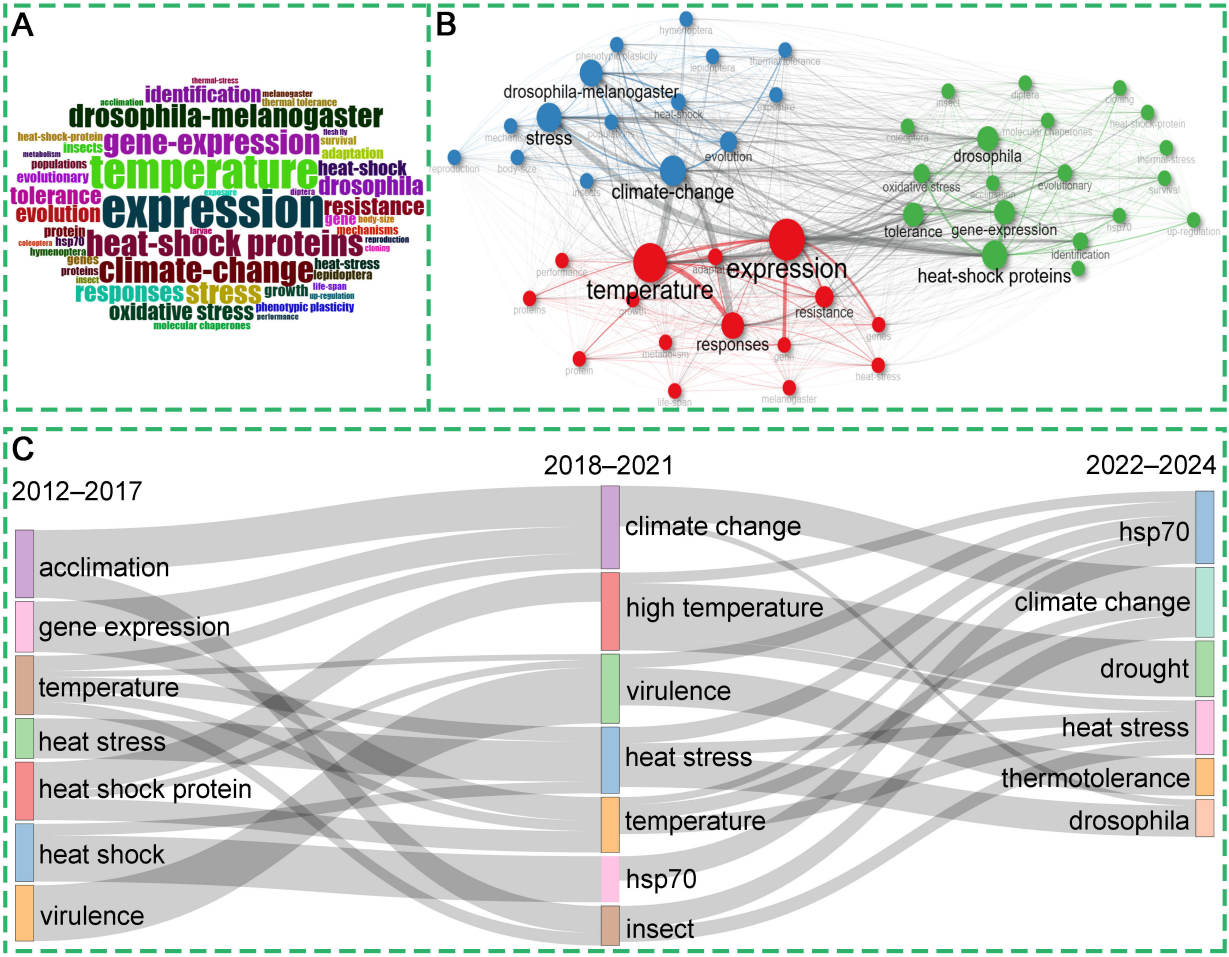
Word cloud co-occurrence network and trending topics of keywords. **(A)** Word cloud of keywords from the insect responses to high temperature studies. **(B)** Keyword co-occurrence network and clusters by Biblioshiny. The nodes represent keywords that meet the filtering threshold. The size of the node is indicative of the frequency of occurrence of the keyword. The curve represents the co-occurrence relationship between the keywords. The color of an element indicates the cluster it belongs to, and different clusters are indicated by different colors. **(C)** Visualization of theme evolution using Biblioshiny, analysis field set as all keyword, weight index set as inclusion index weighted by word occurrences, time slices set as 2 cutting points (2017 and 2021).

To explore the hot topics in this field, we used the Bibliometrix tool to conduct keyword analyses, including word cloud visualization (**Figure 5A**), co-occurrence analysis (**Figure 5B**), and thematic evolution analysis (**Figure 5C**). As shown in **Figure 5A** and **Supplementary Figure S2**, the 10 most frequent keywords consisted of expression, temperature, heat-shock proteins, climate change, gene expression, *drosophila-melanogaster*, stress, responses, *drosophila*, and tolerance. By performing keyword co-occurrence analysis, keywords can be categorized into three groups, as shown in **Figure 5B**. The red cluster represents the adaptation mechanisms and physiological responses of insects under heat-stress conditions. Expression, temperature, responses, tolerance, etc. are the most common keywords, oxidative stress, growth, adaptation, performance and life-span are also frequently mentioned (**Figure 5B**, **Supplementary Table S7**). The cluster explains how insects adapt to and cope with heat stress through mechanisms involving gene expression and protein regulation, including aspects such as oxidative stress, tolerance, metabolic adjustments, growth, and lifespan. The primary keywords of the blue cluster are heat-shock proteins, climate change, evolution, phenotypic plasticity, thermal tolerance, and acclimation (**Figure 5B**, **Supplementary Table S7**). These keywords collectively focus on how insects adapt to climate change and heat stress conditions through heat-shock proteins and evolutionary mechanisms, covering aspects such as phenotypic plasticity, population adaptation, body size changes, reproduction, and population structure. The green cluster includes the terms gene expression, *drosophila-melanogaster*, resistance, identification, heat-shock, evolutionary, hsp70, heat-shock-protein, molecular chaperones, and thermal stress (**Figure 5B**, **Supplementary Table S7**). These keywords collectively point to research on the expression and tolerance mechanisms of heat shock proteins (especially HSP70) in *Drosophila* and other Diptera insects under heat stress conditions, including aspects such as the upregulation of gene expression, the role of molecular chaperones, and the impact of heat stress on survival. As illustrated in **Figure 5C**, the trending topics also align with the findings of the keyword co-occurrence analysis. The most frequent keywords in different years include virulence, gene expression, heat stress, heat shock protein, and hsp70. This result indicates that heat shock proteins play a crucial role in insect response to high temperatures, and HSP70 is one of the most important members among them. Interestingly, since 2018, the term “climate change” has begun to appear and continues to be present (**Figure 5B**). This indicates that researchers are directing their attention towards the impact of climate change on the capacity of insects to adapt to elevated temperatures, as well as the physiological and biological alterations they undergo in response to global warming. This suggests that researchers are increasingly focusing on the effects of climate change on the ability of insects to adapt to elevated temperatures, as well as the physiological and biological changes they undergo in response to global warming. It is also interesting that the word “drought” appears suggesting that researchers are potentially also addressing other environmental factors such as precipitation and humidity. Connected with this, changes in soil temperature and humidity, as well as air quality such as ozone concentrations, occurring in conjunction with high temperatures, continue to require more research. Furthermore, this signifies an increasing acknowledgement of the significance of comprehending the mechanisms through which insects, as integral components of ecosystems, adapt and maintain their survival in the face of global climate change.

In order to gain insight into research frontiers in this field, we analyzed the Sigma values of literature pertaining to insect response to high temperatures (including corresponding references) and summarized the top 10 articles with the highest Sigma values (**Table 1**). The article with the highest sigma value is that of Overgaard (2012), published in the open-access journal PLOS ONE (38) (**Table 1**). The second one is Terblanche JS (2011) which was published in the journal ‘Experimental Biology’ (39) (**Table 1**). It is noteworthy that in 2016, the paper ‘What can plasticity contribute to insect responses to climate change?’ (40) was published. The 2015 paper ‘Stage-specific heat effects: timing and duration of heat waves alter demographic rates of a global insect pest.’ (41) and the 2014 paper ‘Strong negative effects of simulated heat waves in a tropical butterfly.’ (42) (**Table 1**). Despite their published relatively late, these papers have achieved high Sigma values, indicating that the emergence of disasters such as climate change and heat waves has attracted significant attention within the scientific community. Furthermore, the research presented in these papers reflects the current research frontier and hotspot in this field, namely the urgent need for insects to respond to climate change and heat waves.

**Table 1:** Sigma analysis of the papers on insect responses to high temperature.

Rank	Document Doi	Author	Publication year
1	Validity of thermal ramping assays used to assess thermal tolerance in arthropods.	Overgaard et al. (38)	2012
2	Ecologically relevant measures of tolerance to potentially lethal temperatures.	Terblanche et al. (39)	2011
3	What can plasticity contribute to insect responses to climate change?	Sgrò et al. (40)	2016
4	Stage-specific heat effects: timing and duration of heat waves alter demographic rates of a global insect pest.	Zhang et al. (41)	2015
5	Strong negative effects of simulated heat waves in a tropical butterfly.	Fischer et al. (42)	2014
6	Thermal ramping rate influences evolutionary potential and species differences for upper thermal limits in Drosophila.	Mitchell et al. (43)	2010
7	Heat shock factors: integrators of cell stress, development and lifespan.	Åkerfeltl et al. (44)	2010
8	Heat shock reduces stalled RNA polymerase II and nucleosome turnover genome-wide.	Teves et al. (45)	2011
9	Cloning and expression of five heat shock protein genes in relation to cold hardening and development in the leafminer, Liriomyza sativa.	Huang et al. (46)	2009
10	Molecular characterization of heat shock protein 90, 70 and 70 cognate cDNAs and their expression patterns during thermal stress and pupal diapause in the corn earworm.	Zhang et al. (47)	2010

**Figure 6:**
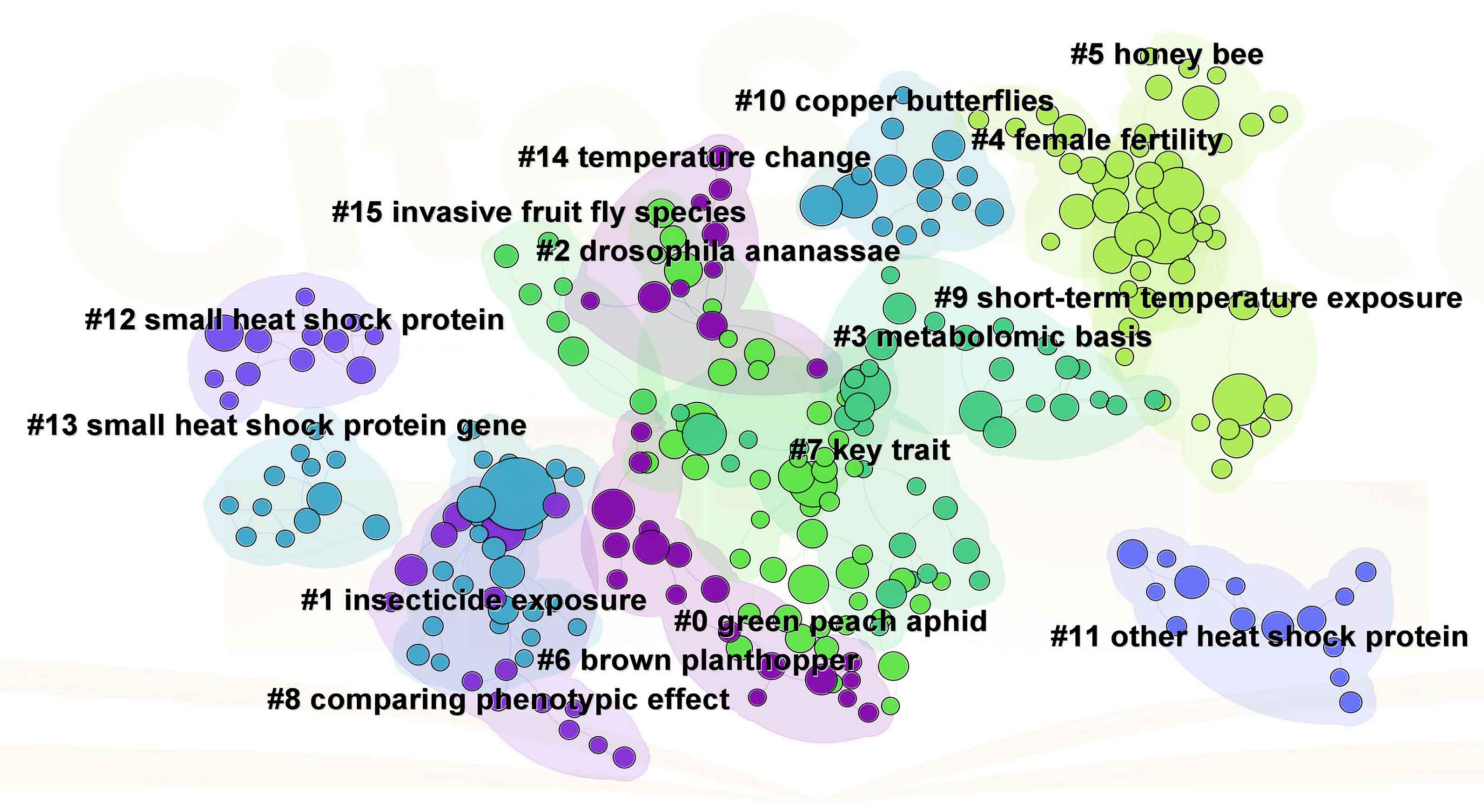
Bibliographic coupling reference map of research on insect responses to high temperature. Bibliographic coupling reference network with clustering visualization. The size of each node indicates the citation of each reference. Each color represents a clustering situation.

Furthermore, to explore the research evolution in the field of insect responses to high temperatures, we undertook a citation bibliographic coupling analysis of the most frequently cited references. A complex bibliographic coupling network of references was constructed by using CiteSpace software, as illustrated in **Figure 6**, identifying fifteen clusters across all references, thus offering a perspective that extends beyond the mere occurrence of keywords. The largest cluster is the #0 green peach aphid, with an S value of 0.949. The article by Chen et al. (2022), represents the key reference within this cluster (**Figure 6**, **Table 2**), which suggests that it has a relatively high impact or potential influence within the context of the “green peach aphid” cluster. In addition to the #0 green peach aphid, a clustering group of #2 *Drosophila ananassae*, #5 Honey bee, #6 Brown planthopper, and #10 Copper butterflies were also discovered (**Figure 6**, **Table 2**). These insects serve as crucial models for research in genetics and evolutionary biology, social behavior and neurobiology, agricultural pest resistance and ecology, as well as evolutionary biology and ecology (48–50). Among these cluster, #6 Brown planthopper has a high frequency of 140, which is likely because it is a hotspot for agricultural pests and its study has received much attention. Researchers in multiple fields are committed to delving deeper into the biology and behavioral mechanisms of brown planthopper in order to better address the challenges of this agricultural pest. #10 copper butterflies have a frequency of 115, indicating strong research interest in their ecological roles and conservation. With respect to the study of insect responses to high temperatures, chronologically research attention has shifted from an emphasis on #8 comparing phenotypic effect toward an investigation of #7 key trait, #3 metabolomic basis and #4 female fertility (**Figure 6**, **Table 2**), a transition marks a shift in focus from the description of macroscopic characteristics to an exploration of the biochemical and reproductive mechanisms occurring at the micro-level, aiming to uncover biological responses and adaptive strategies under heat stress. Interestingly, the clustering of #1 insecticide exposure was also observed (**Figure 6**, **Table 2**), which may suggest a potential synergistic effect or interaction between high-temperature exposure and insecticide exposure, or possibly a shared response mechanism. This discovery not only reveals the multiple environmental pressures faced by insects but also suggests that in the context of climate warming, we may need to reassess and adjust insecticide use strategies and dosages.

**Table 2:** Main contents of the cluster analysis of papers on insect responses to high temperature from 1978 to 2022.

Group project	Group cocitation	Mean Year	The major citing article	Frequency	The most cited article
#0	green peach aphid	2018	10.1016/j.aspen.2022.101992	2	10.1111/1744-7917.12505.
#1	insecticide exposure	2012	10.1093/jisesa/ieu032	24	10.1146/annurev-ento-011613-162107
#2	drosophila ananassae	2017	10.1111/ele.14421	0	10.1111/1365-2435.13279
#3	metabolomic basis	2016	10.1111/gcb.13407	115	10.1146/annurev-ento-010814-021017
#4	female fertility	2019	10.1016/j.jinsphys.2023.104491	5	10.1111/brv.12588
#5	honey bee	2019	10.3389/fphys.2023.1251235	2	10.1038/s41467-018-07273-z
#6	brown planthopper	2009	10.1111/j.1365-2435.2011.01898.x	140	10.1111/j.1742-4658.2009.07470.x
#7	key trait	2015	10.1111/jeb.12832	41	10.18637/jss.v067.i01
#8	comparing phenotypic effect	2008	10.1016/j.jinsphys.2012.01.016	72	10.1093/acprof:oso/9780198570875.001.1
#9	short-term temperature exposure	2020	10.1016/j.jinsphys.2023.104491	5	10.1146/annurev-ento-041520-074454
#10	copper butterflies	2013	10.1111/gcb.13407	115	10.1111/j.1365-2435.2012.02036.x

## 4 Discussion

In this study, a comprehensive bibliometric analysis and content analysis of 4,417 papers related to insect responses to high temperatures, published in the Web of Science Core Collection database, was conducted using CiteSpace, VOSviewer software, and the Bibliometrix package. Firstly, the fundamental aspects of the field are described through the application of basic statistical analysis, including publication time, publication volume, main journals, academic categories, and the breadth of research. Subsequently, through collaboration analysis was employed to elucidate the international and inter-institutional cooperation dynamics within the field, identifying which countries, institutions, and authors are more inclined to engage in cooperation and exchange. Ultimately, the utilization of co-citation and co-occurrence analysis enables the identification of advancements within the field and highlights research topics that receive greater attention, thereby elucidating the evolutionary dynamics that characterize the field. Furthermore, this study identifies potential future research hotspots or trends, thereby establishing a comprehensive knowledge framework that will facilitate further development of the field.

The analysis of keywords and references in terms of their co-occurrence allows for the understanding of the research trends and current hotspots, which is of great significance in the prediction of future research dynamics (24, 25, 29). In this study, the most frequently occurring keywords were temperature, gene-expression, heat-shock proteins, climate-change, and *Drosophila melanogaster*, which represent the current research hotspots and frontiers. The co-occurring category is largely consistent with the overall statistical profile, concentrating on gene-expression, heat-shock proteins, and climate-change. Climate change is now thought to be a factor in the decline of insect populations worldwide (7, 51). Forecasting the responses of insects to climate change may now be a major focus of research in this area Currently, the journal Current Opinion in Insect Science publishes many papers on the effects of climate change on insects and has established dedicated research topics around this theme (7, 52, 53). Additionally, clusters in collaborations and co-citations related to climate change demonstrate that it is a hot research focus. It is expected that an ever-increasing proportion of researchers and scholars will devote their attention to the subject of insect adaptation to climate change. Specifically, Inferences based on the combination of keywords and Current Opinion in Insect Science suggest that future research may concentrate on the immunity and adaptation of insects to climate change. For example, the effect of rising temperatures on insect gut health and the interactions between insects and their associated microorganisms (7), epigenetic effects of climate change on insects (54), Climate change causes insect community shifts and population explosions (55), ecological projections of insect distribution dynamics under climate change (56), and genome complexities modulate insect response to climate change, etc. (53). Additionally, phenotypic plasticity is one of the nongenetic strategies used by insects to cope with environmental variation (2). In this work, the words “phenotypic plasticity” showed high frequency. Insects can respond by morphological responses to high environmental variation (2). However, the increase in heat resistance does not come without a cost, and it triggers a series of concurrent costs associated with a range of other plasticity properties related to adaptability. For example, frequent heat exposure sometimes leads to produces larger insect individuals. The selective advantages originally associated with smaller body sizes may become unfavorable (2). Additionally, a high frequency of “gene expression” and “expression”, was observed. With advancements in molecular technology, transcriptome sequencing is becoming more routine. It can focus on the gene expression level, accurately capture which genes are activated or repressed in high temperature environment, and dynamically reflect the changes in gene expression, which provides a powerful support for the in-depth understanding of the mechanism of high-temperature adaptation in insects. Heat shock proteins are known to be proteins that respond to high temperature stress. It acts as a molecular chaperone, promoting the correct refolding of denatured proteins and preventing aggregation under heat stress (57, 58). The expression of heat shock protein genes is rapidly up-regulated when insects are exposed to high temperatures, a process that can be sensitively captured by transcriptome studies and can give us a clear picture of the immediate molecular responses of insects to high temperatures (20, 21, 36, 59). Heat shock proteins are the most abundant and most studied class of stress proteins. Our findings indicate that heat shock proteins were an important topic of investigation throughout the period under review (2012–2024), as research is ongoing, have been identified in a wide range of heat shock proteins to HSP70 functions (11) (**Figure 5**). It is therefore our contention that HSP remain the same a significant research theme in the study of future insect responses to climate change. Furthermore, the results of both keyword clustering and reference co-citation clustering indicated that *Drosophila* was grouped into a single category. The genus *Drosophila* represents an ideal model for investigating evolutionary responses to extreme temperatures, offering a diverse array of thermal niches and habitat requirements. This genus encompasses species with narrow and restricted distributions, as well as more widely-ranging cosmopolitan species (37). Therefore, many future studies will utilize the *Drosophila* model to further investigate insect adaptations in the context of climate change.

Overall, a systematic literature review on the high-temperature stress of insects was conducted in this study. Although high-temperature stress of insects has been thoroughly studied, we pointed out some questions that are still without answers, which could be tackled in upcoming research. For example, the integrated response of insects to high temperatures involves mechanisms such as perception and response. However, mechanisms such as metabolic changes, thermal coma, stress response, and thermoregulation, in addition to heat shock proteins, have been understudied in non-model insects. In addition, global warming can interact with other types of disasters, such as droughts, extreme rainfall, floods, and fires, and can even synergize with pollution stressors like pesticides. These combined effects can exacerbate the stresses imposed on insects. Phenotypic plasticity and evolutionary adaptation are potential evolutionary mechanisms that insects may use to cope with ongoing climate change. However, further exploration of these mechanisms is necessary, given that the available evidence is inconclusive, including transgenerational effects, and there is insufficient evidence of insect adaptation to global warming.

## 5 Conclusion

Here, we use a bibliographical approach to conduct a comprehensive quantitative assessment of global research priorities and trends on the effects of high-temperature stress on insects. Utilizing the Web of Science Core Collections database was employed to identify and collate a total of 4417 papers, comprising 4094 articles and 323 review papers. Then we used the CiteSpace and VOSviewer software and Bibliometrix package to examine all articles concerning research on insect responses to high temperatures.

The results of the analysis are summarized as follows (1): Since 2012, the number of documents published in the field of insect responses to high temperatures has exhibited explosive growth, with an average annual growth rate of 3.59%. This outcome serves to illustrate the growing significance of this research category on a global scale. It is anticipated that the continuous growth in the number of publications will significantly enhance the scientific understanding of the researchers engaged in this field of study. (2) According to a statistical analysis PLoS One is the most productive journal and also the most influential, with a high h-index of 41. On the other hand, the PNAS have a significant impact as well, with only 33 published articles, yet they have the highest citation with an h-index of 26. Hoffmann AA and Du YZ are the most productive authors, with Hoffmann AA ranked as the top author globally in terms of g-index, h-index and total citations. Among the top 10 most influential countries in this field—China, the United States, India, Brazil, Germany, the United Kingdom, Spain, Canada, Japan, and Australia, respectively. Of these, China, India, and Brazil are developing countries. The China and USA are the most active countries, with high publications, citations, and total link strengths. It is interesting to note that Southeast Asian countries such as Japan and Korea, as well as developing countries such as Brazil and India, are collaborators with the United States. In contrast, China works more with developed European countries. China is a strong performer in terms of productivity, but like other developing countries, it needs to improve both productivity and global cooperation. The Chinese Academy of Agricultural Sciences, the University of California system, and the Centre National De La Recherche Scientifique (CNRS) are the leading institutions. Although the Chinese Academy of Agricultural Sciences has the highest number of publications, it has the lowest centrality, suggesting that its international cooperation needs to be deepened. (3) Gene expression, temperature, heat-shock proteins, climate change, *drosophila-melanogaster*, and tolerance are the most frequent keywords. All the keywords could be clustered into three categories, each about the adaptation mechanisms and physiological responses of insects under heat-stress conditions, how insects adapt to climate change and heat-stress conditions through heat-shock proteins and evolutionary mechanisms and the expression and tolerance mechanisms of heat shock proteins in *Drosophila* and other Diptera insects under heat stress conditions topics, respectively. Cluster analysis of bibliographic coupling identified 15 major research themes, results demonstrate that the field evolved from describing physiological traits to dissecting biochemical and reproductive mechanisms underlying thermal tolerance, aiming to uncover biological responses and adaptive strategies under heat stress. By combining keyword clustering and bibliographic coupling, we observe that the newer clusters are more focused on these detailed mechanistic studies, suggesting that the field is moving towards a more in-depth exploration of the biological processes involved in thermal tolerance.

This paper represents the inaugural comprehensive analysis of high-temperature stress on insect research, acknowledging the significant contributions of prominent authors, academic institutions, and research networks. In summary, further understanding of the mechanisms behind high-temperature stress on insects not only helps address the risks posed by climate change but also contributes to promoting agricultural productivity, protecting biodiversity, and developing more effective insect management strategies.
