# Supplementary material for: Research progress and trends of insect high-temperature stress: insights from bibliometric analysis
Source: Front Insect Sci. 2025 Jul 30;5:1625155. doi: 10.3389/finsc.2025.1625155 (PMC12343620; doi:10.3389/finsc.2025.1625155)
Supplement: Supplementary file 1 [file Supplementaryfile1.docx]

Supplementary Material

## Supplementary Figures


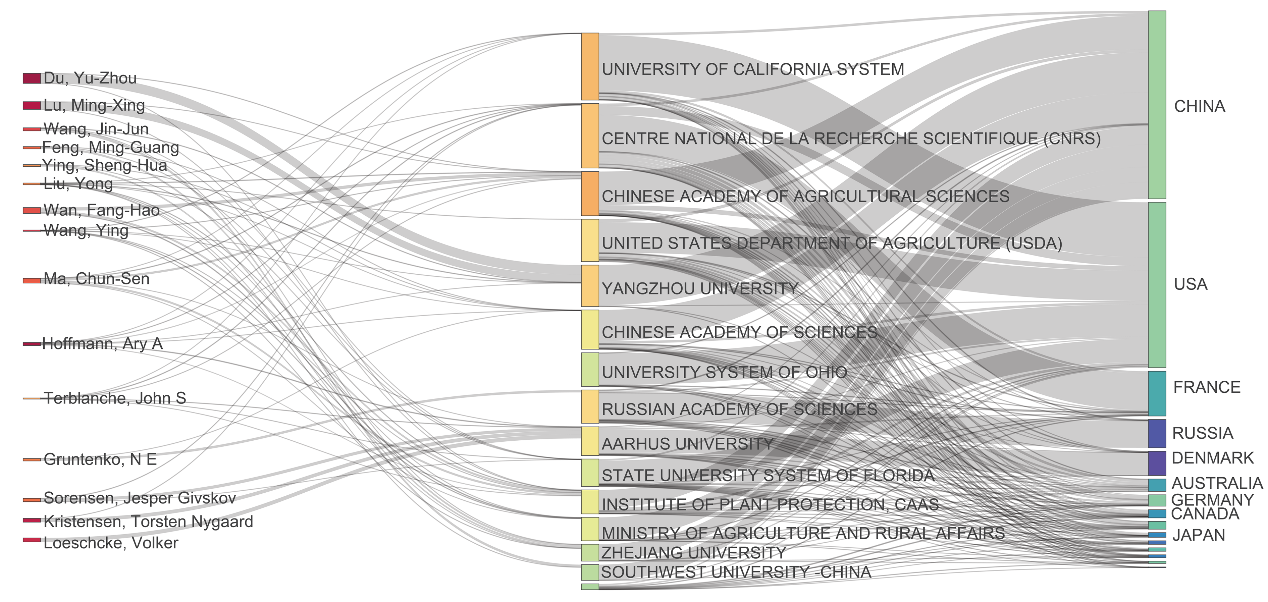


**Supplementary Figure S1.** A Sankey diagram depicting the collaborative relationships among corresponding authors, their affiliated institutions, and countries.


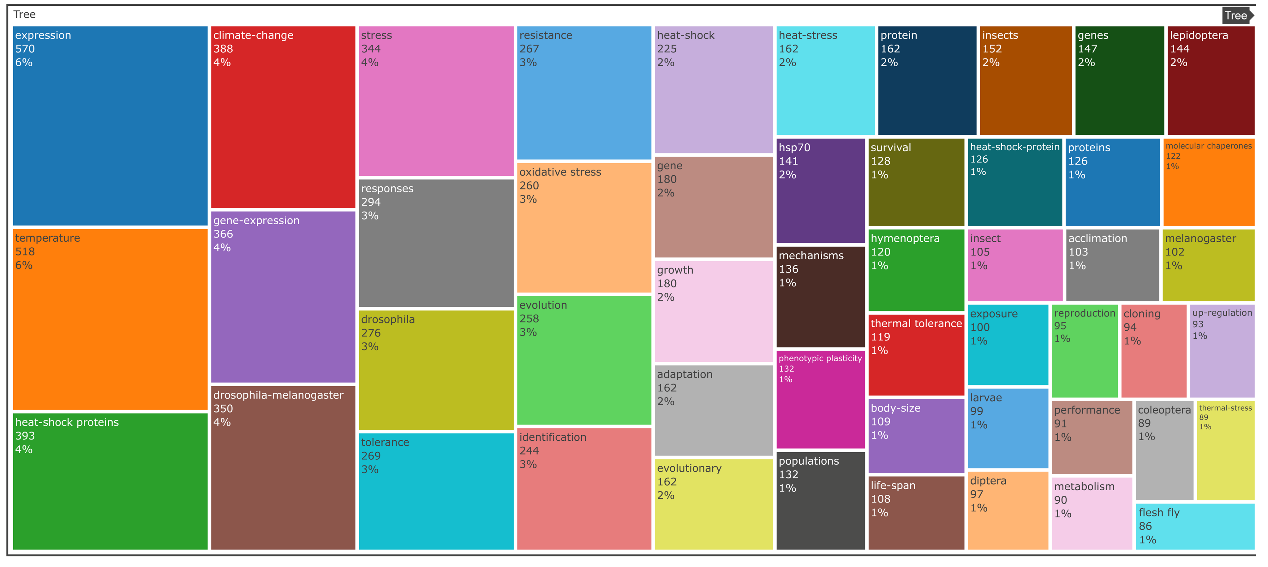


**Supplementary Figure S2.** Treemap of top 50 keyword with the percentage of occurrences by Biblioshiny.

## Supplementary Tables

| **Table S1 The most relevant source information between 2012-2024 in papers relating to insect responses to high temperature.** | | | | | | |
| --- | --- | --- | --- | --- | --- | --- |
| **Source** | **h_index** | **g_index** | **m_index** | **TC** | **NP** | **cited** |
| PLOS ONE | 41 | 61 | 3.154 | 5604 | 208 | 6397 |
| JOURNAL OF INSECT PHYSIOLOGY | 31 | 45 | 2.385 | 2452 | 94 | 6177 |
| JOURNAL OF EXPERIMENTAL BIOLOGY | 29 | 43 | 2.231 | 2080 | 71 | 3837 |
| SCIENTIFIC REPORTS | 28 | 42 | 2.333 | 2469 | 125 | 2508 |
| PROCEEDINGS OF THE NATIONAL ACADEMY OF SCIENCES OF THE UNITED STATES OF AMERICA | 26 | 33 | 2.000 | 2041 | 33 | 7187 |
| JOURNAL OF BIOLOGICAL CHEMISTRY | 20 | 28 | 1.538 | 1190 | 28 | 4016 |
| CELL STRESS & CHAPERONES | 20 | 31 | 1.538 | 1140 | 57 | 1613 |
| JOURNAL OF THERMAL BIOLOGY | 18 | 27 | 1.385 | 1158 | 108 | 2223 |
| BMC GENOMICS | 18 | 29 | 1.385 | 903 | 39 | 1203 |
| FUNCTIONAL ECOLOGY | 17 | 32 | 1.308 | 1974 | 32 | 2425 |
| MOLECULAR ECOLOGY | 17 | 25 | 1.308 | 890 | 25 | 1127 |
| JOURNAL OF EVOLUTIONARY BIOLOGY | 17 | 25 | 1.308 | 639 | 28 | 1561 |
| SCIENCE OF THE TOTAL ENVIRONMENT | 17 | 24 | 1.889 | 622 | 30 | 656 |
| PHYSIOLOGICAL ENTOMOLOGY | 16 | 28 | 1.231 | 835 | 37 | 984 |
| INSECT SCIENCE | 16 | 27 | 1.231 | 786 | 39 | 35 |
| PLOS GENETICS | 15 | 18 | 1.154 | 829 | 18 | 1093 |
| FRONTIERS IN PHYSIOLOGY | 15 | 22 | 1.154 | 669 | 59 | 530 |
| JOURNAL OF ECONOMIC ENTOMOLOGY | 15 | 22 | 1.154 | 646 | 55 | 2045 |
| COMPARATIVE BIOCHEMISTRY AND PHYSIOLOGY A-MOLECULAR & INTEGRATIVE PHYSIOLOGY | 15 | 23 | 1.154 | 640 | 23 | 1017 |
| ECOLOGY AND EVOLUTION | 15 | 23 | 1.154 | 604 | 40 | 1194 |
| APPLIED MICROBIOLOGY AND BIOTECHNOLOGY | 15 | 21 | 1.364 | 559 | 21 | 410 |
| INSECTS | 14 | 23 | 2.000 | 783 | 104 | 801 |
| CURRENT OPINION IN INSECT SCIENCE | 14 | 21 | 1.400 | 744 | 21 | 679 |
| PLOS NEGLECTED TROPICAL DISEASES | 14 | 25 | 1.077 | 628 | 28 | 638 |
| INSECT MOLECULAR BIOLOGY | 14 | 22 | 1.077 | 511 | 31 | 1407 |
| MOLECULAR CELL | 13 | 13 | 1.000 | 1341 | 13 | 1 |
| FRONTIERS IN PLANT SCIENCE | 13 | 30 | 1.000 | 951 | 30 | 870 |
| ENVIRONMENTAL ENTOMOLOGY | 13 | 19 | 1.000 | 473 | 45 | 1 |
| PEST MANAGEMENT SCIENCE | 13 | 20 | 1.182 | 461 | 37 | 934 |
| GENE | 13 | 20 | 1.000 | 436 | 27 | 757 |

| **Table S2 Average citations per publication and relevant information of the top twenty published countries** | | | | | | | |
| --- | --- | --- | --- | --- | --- | --- | --- |
| **Country** | **Articles** | **Articles %** | **SCP** | **MCP** | **MCP %** | **TC** | **Average Article Citations** |
| CHINA | 1129 | 25.6 | 920 | 209 | 18.5 | 16452 | 14.60 |
| USA | 796 | 18 | 611 | 185 | 23.2 | 24964 | 31.40 |
| INDIA | 207 | 4.7 | 167 | 40 | 19.3 | 3054 | 14.80 |
| BRAZIL | 157 | 3.6 | 119 | 38 | 24.2 | 1970 | 12.50 |
| GERMANY | 156 | 3.5 | 106 | 50 | 32.1 | 4749 | 30.40 |
| UNITED KINGDOM | 145 | 3.3 | 65 | 80 | 55.2 | 4444 | 30.60 |
| SPAIN | 126 | 2.9 | 84 | 42 | 33.3 | 2866 | 22.70 |
| CANADA | 115 | 2.6 | 86 | 29 | 25.2 | 3162 | 27.50 |
| JAPAN | 114 | 2.6 | 87 | 27 | 23.7 | 1833 | 16.10 |
| AUSTRALIA | 112 | 2.5 | 75 | 37 | 33 | 2987 | 26.70 |
| FRANCE | 102 | 2.3 | 54 | 48 | 47.1 | 2303 | 22.60 |
| RUSSIA | 98 | 2.2 | 86 | 12 | 12.2 | 991 | 10.10 |
| KOREA | 90 | 2 | 65 | 25 | 27.8 | 1250 | 13.90 |
| ITALY | 89 | 2 | 63 | 26 | 29.2 | 1739 | 19.50 |
| DENMARK | 68 | 1.5 | 39 | 29 | 42.6 | 1724 | 25.40 |
| POLAND | 65 | 1.5 | 53 | 12 | 18.5 | 1225 | 18.80 |
| BELGIUM | 64 | 1.4 | 31 | 33 | 51.6 | 1756 | 27.40 |
| SOUTH AFRICA | 50 | 1.1 | 25 | 25 | 50 | 980 | 19.60 |
| MEXICO | 47 | 1.1 | 37 | 10 | 21.3 | 785 | 16.70 |
| IRAN | 42 | 1 | 35 | 7 | 16.7 | 473 | 11.30 |

| **Table S3 Top 10 institutions and author in the field of insect responses to high temperature.** | | | | | | | | | | | |
| --- | --- | --- | --- | --- | --- | --- | --- | --- | --- | --- | --- |
| **Rank** | **Institution** | **Articles** | **Centrality** | **Country** | **Rank** | **Author** | **Articles** | **h_index** | **g_index** | **m_index** | **TC** |
| 1 | CHINESE ACADEMY OF AGRICULTURAL SCIENCES | 200 | 0.05 | CHINA | 1 | Hoffmann, AA | 43 | 22 | 43 | 1.69 | 2174 |
| 2 | UNIVERSITY OF CALIFORNIA SYSTEM | 195 | 0.33 | USA | 2 | Ma, CS | 26 | 18 | 28 | 1.39 | 988 |
| 3 | CENTRE NATIONAL DE LA RECHERCHE SCIENTIFIQUE (CNRS) | 176 | 0.19 | FRANCE | 3 | Du, YZ | 46 | 15 | 24 | 1.25 | 700 |
| 4 | YANGZHOU UNIVERSITY | 170 | 0.04 | CHINA | 4 | Loeschcke, V | 29 | 15 | 25 | 1.15 | 644 |
| 5 | RUSSIAN ACADEMY OF SCIENCES | 159 | 0.05 | RUSSIAN | 5 | Colinet, H | 21 | 14 | 21 | 1.08 | 902 |
| 6 | UNITED STATES DEPARTMENT OF AGRICULTURE (USDA) | 158 | 0.11 | USA | 6 | Feng, MG | 29 | 14 | 26 | 1.08 | 724 |
| 7 | AARHUS UNIVERSITY | 130 | 0.08 | DENMARK | 7 | Lu, MX | 35 | 14 | 23 | 1.17 | 631 |
| 8 | CHINESE ACADEMY OF SCIENCES | 127 | 0.11 | CHINA | 8 | Morcillo, G | 14 | 14 | 14 | 1.08 | 548 |
| 9 | INSTITUTE OF PLANT PROTECTION, CAAS | 116 | 0.17 | CHINA | 9 | Overgaard, J | 21 | 14 | 21 | 1.08 | 962 |
| 10 | MINISTRY OF AGRICULTURE AND RURAL AFFAIRS | 116 | 0.07 | CHINA | 10 | Xu, BH | 22 | 13 | 22 | 1.00 | 504 |

| **Table S4. Author co-citation.** | | |
| --- | --- | --- |
| Author | Citations | Total link strength |
| Hoffmann, AA | 1009 | 23049 |
| Sorensen, JG | 687 | 15354 |
| Colinet, H | 626 | 14962 |
| Chown, SL | 487 | 12691 |
| Sinclair, BJ | 427 | 12117 |
| Overgaard, J | 395 | 11706 |
| Kingsolver, JG | 517 | 11641 |
| Huey, RB | 492 | 11361 |
| Feder, ME | 532 | 10444 |
| Angilleta, MJ | 431 | 10050 |
| Rinehart, JIP | 451 | 9922 |
| Trblanche, JS | 356 | 9619 |
| Kostal, V | 354 | 9487 |
| Krebs, RA | 353 | 7976 |
| Macmillan, HA | 221 | 7039 |

| **Table S5. journals co-citation.** | | |
| --- | --- | --- |
| Source | Citations | Totalik strength |
| Proceedings of the National Academy of Sciences of the United States of America | 7187 | 510975 |
| PLoS One | 6403 | 416857 |
| Journal of Insect Physiology | 6177 | 347816 |
| Nature | 4595 | 338477 |
| Science | 4621 | 337987 |
| Journal of Biological Chemistry | 4018 | 292829 |
| Journal of Experimental Biology | 3837 | 242774 |
| Cell | 3044 | 235629 |
| Scientific Reports | 2508 | 180284 |
| Functional Ecology | 2428 | 156455 |
| Annual Review of Entomology | 2485 | 153581 |
| Proceedings of the Royal Society B-Biological Sciences | 2222 | 148721 |
| Nucleic Acids Research | 2277 | 148573 |
| Journal of Thermal Biology | 2225 | 131649 |
| Genetics | 1854 | 130140 |

| **Table S5. Reference co-citation.** | | |
| --- | --- | --- |
| Cited reference | Citations | Total link strength |
| Heat-shock proteins, molecular chaperones, and the stress response: evolutionary and ecological physiology. | 395 | 6011 |
| Adaptation of *Drosophila* to temperature extremes: Bringing together quantitative and molecular approaches. | 223 | 4249 |
| The evolutionary and ecological role of heat shock proteins | 241 | 4213 |
| Thermal Adaptation: A Theoretical and Empirical Synthesis | 232 | 4106 |
| Insect heat shock proteins during stress and diapause | 258 | 4069 |
| Analysis of relative gene expression data using real-time quantitative PCR and the 2(-Delta Delta C(T)) Method | 439 | 3957 |
| Mechanoenzymatics of titin kinase | 221 | 3906 |
| Up-regulation of heat shock proteins is essential for cold survival during insect diapause | 191 | 3553 |
| Seasonal and Sexual Differences in the Dorsal Skin Gland of the Kangaroo Rat (*Dipodomys*) | 352 | 3279 |
| Insect thermal tolerance: what is the role of ontogeny, ageing and senescence? | 142 | 2718 |
| Upper thermal limits in terrestrial ectotherms: how constrained are they? | 119 | 2417 |
| Insects in fluctuating thermal environments | 140 | 2381 |
| Enhancing the protein production levels in Escherichia coli with a strong promoter | 117 | 2276 |
| Ecologically relevant measures of tolerance to potentially lethal temperatures | 110 | 2274 |
| Thermal tolerance, climatic variability and latitude | 104 | 2087 |

| **Table S7 Main keywords involved in clustering and clustering information** | | | | |
| --- | --- | --- | --- | --- |
| **Node** | **Cluster** | **Betweenness** | **Closeness** | **PageRank** |
| gene-expression | 1 | 9.306 | 0.019 | 0.031 |
| drosophila-melanogaster | 1 | 11.915 | 0.020 | 0.035 |
| resistance | 1 | 6.179 | 0.020 | 0.029 |
| identification | 1 | 2.934 | 0.019 | 0.020 |
| heat-shock | 1 | 3.537 | 0.019 | 0.020 |
| evolutionary | 1 | 3.597 | 0.019 | 0.024 |
| hsp70 | 1 | 2.123 | 0.019 | 0.019 |
| survival | 1 | 1.743 | 0.019 | 0.016 |
| heat-shock-protein | 1 | 0.914 | 0.016 | 0.013 |
| molecular chaperones | 1 | 1.022 | 0.016 | 0.015 |
| exposure | 1 | 0.759 | 0.017 | 0.012 |
| larvae | 1 | 0.978 | 0.019 | 0.012 |
| diptera | 1 | 0.404 | 0.017 | 0.010 |
| cloning | 1 | 0.728 | 0.016 | 0.013 |
| up-regulation | 1 | 0.777 | 0.016 | 0.014 |
| thermal-stress | 1 | 0.867 | 0.017 | 0.012 |
| flesh fly | 1 | 0.515 | 0.015 | 0.012 |
| expression | 2 | 24.870 | 0.020 | 0.051 |
| temperature | 2 | 25.693 | 0.020 | 0.051 |
| responses | 2 | 7.897 | 0.020 | 0.035 |
| tolerance | 2 | 11.587 | 0.020 | 0.035 |
| oxidative stress | 2 | 5.381 | 0.019 | 0.023 |
| gene | 2 | 1.284 | 0.016 | 0.015 |
| growth | 2 | 1.358 | 0.017 | 0.014 |
| adaptation | 2 | 2.783 | 0.019 | 0.023 |
| heat-stress | 2 | 1.608 | 0.017 | 0.016 |
| protein | 2 | 0.700 | 0.016 | 0.013 |
| insects | 2 | 1.629 | 0.019 | 0.016 |
| genes | 2 | 1.514 | 0.018 | 0.016 |
| mechanisms | 2 | 1.747 | 0.018 | 0.016 |
| proteins | 2 | 0.588 | 0.016 | 0.013 |
| life-span | 2 | 0.555 | 0.015 | 0.012 |
| melanogaster | 2 | 0.768 | 0.016 | 0.012 |
| performance | 2 | 0.571 | 0.017 | 0.012 |
| metabolism | 2 | 0.657 | 0.017 | 0.011 |
| heat-shock proteins | 3 | 10.236 | 0.018 | 0.037 |
| climate-change | 3 | 6.792 | 0.019 | 0.037 |
| stress | 3 | 12.918 | 0.019 | 0.038 |
| drosophila | 3 | 11.157 | 0.020 | 0.033 |
| evolution | 3 | 4.590 | 0.019 | 0.024 |
| lepidoptera | 3 | 2.281 | 0.019 | 0.017 |
| phenotypic plasticity | 3 | 0.670 | 0.016 | 0.017 |
| populations | 3 | 1.090 | 0.018 | 0.016 |
| hymenoptera | 3 | 0.723 | 0.016 | 0.012 |
| thermal tolerance | 3 | 1.584 | 0.018 | 0.017 |
| body-size | 3 | 0.485 | 0.016 | 0.013 |
| insect | 3 | 1.039 | 0.018 | 0.013 |
| acclimation | 3 | 1.192 | 0.018 | 0.017 |
| reproduction | 3 | 0.552 | 0.016 | 0.011 |
| coleoptera | 3 | 0.204 | 0.016 | 0.009 |
